# Dr. R. W. Morgan Retired

**Published:** 1902-01

**Authors:** 


### DR. R. W. MORGAN RETIRED.

Upon the expiration of his "sick leave" Dr. Morgan, of the Army Dental Examining Board, will retire from the service.

Dr. John H. Hess, Army Dental Surgeon, now stationed at West Point, has been promoted to the position.

Dr. Hess is a young man about thirty years of age and reported to possess rather more than the average foundation knowledge of the work of his profession.

